# Adenoid Cystic Carcinoma of the Oral Cavity- A Case Report Comparing the Differences and Similarities Between a Child and an Adult Patient

**DOI:** 10.7759/cureus.39056

**Published:** 2023-05-15

**Authors:** Srinidhi Kasthurirengan, Mahathi Neralla, Senthil Murugan Pandurangan

**Affiliations:** 1 Oral and Maxillofacial Surgery, Saveetha Dental College And Hospitals, Chennai, IND; 2 Oncology, Saveetha Dental College And Hospitals, Chennai, IND

**Keywords:** pediatric salivary gland tumour, pediatric pathology, adenoid cystic carcinoma, buccal mucosa, benign and malignant neoplasm, : salivary gland tumours

## Abstract

Adenoid cystic carcinoma is a malignant neoplasm primarily of the salivary gland, which can also involve lacrimal glands and other exocrine glands. Adenoid cystic carcinoma rarely presents in the buccal mucosa and young children, and among the major salivary glands, it rarely occurs in the sublingual gland. We are presenting two cases of Grade 1- adenoid cystic carcinoma. One in the buccal mucosa of an eight-year-old boy and another in the sublingual gland of a 50-year-old female patient. The site and age of occurrence can make a huge difference in diagnosis and treatment planning due to the unpredictability of the lesion. Proper diagnosis, treatment planning, and appropriate treatment help improve the lesion's prognosis. Even though such lesions rarely occur, awareness among the Oral and maxillofacial fraternity is very important in providing proper patient care.

## Introduction

Adenoid cystic carcinoma (ACC) is a slow-growing and relentless salivary gland malignancy composed of epithelial and myoepithelial neoplastic cells that forms various patterns, including tubular, cribriform, and solid forms [1]. It is a malignant salivary gland neoplasm. It most commonly occurs in the minor and major salivary glands but also in the lacrimal glands and exocrine glands. It accounts for about 1% of all head and neck cancers and < 10% of all salivary gland tumors. It is the most prevalent tumor of the minor salivary glands and the second most common malignancy of the major salivary glands [2]. Its incidence is slightly higher in females than in males. In Adenoid cystic carcinoma, the deletion of chromosome 1p35-36 is a specific chromosomal trait. It can also be due to an abnormality in the fusion of MYB or MYBL1 oncogenes [3]. 

The salivary gland that is most commonly involved is the parotid gland, submandibular gland, and accessory salivary glands of the palate and tongue. Adenoid cystic carcinoma is an indolent tumor with a persistent and recurrent growth pattern with late onset of distant metastases and death. Compared to other epithelial malignancies with a poor prognosis, adenoid cystic carcinoma has a good 5-year survival rate, but the 10- and 20-year survival rates are notably low [4].

The tumor usually presents with swelling and may have pain and numbness. The perineural spread that causes local recurrence is the most striking aspect of adenoid cystic carcinoma, and its late-onset distant metastasis requires long-term monitoring in every patient [5]. It most commonly occurs in the fifth to sixth decade of life, and the median age of occurrence is 57 years [2]. 

In this case report, we present one case of histologically proven grade 1 adenoid cystic carcinoma of buccal mucosa in an eight-year-old boy, which is a very uncommon presentation occurring in the first decade of life, and another case of the same histological grading of adenoid cystic carcinoma of the sublingual gland (which is not a very common subsite) in a fifty-year-old female patient. The intraoral site predilection for ACC is the hard palate, and in our first case, the subsite affected was buccal mucosa. This occurrence is very uncommon due to its unusual age and subsite presentation. The only clinical sign that could possibly implicate adenoid cystic carcinoma, in this case, was the patient's history of a slow-growing swelling. The latter case was presented in a fifty-year-old female patient, which is typical, but the unusual thing is that it occurred in the sublingual salivary gland.

## Case presentation

Case 1

**Figure 1:**
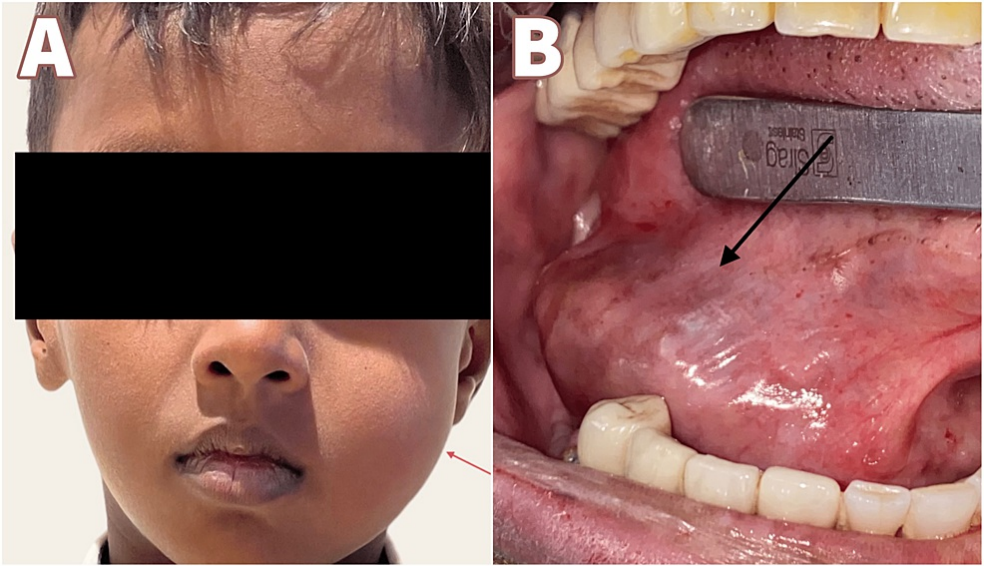
Preoperative image of the lesion. In the above image, 1A depicts a preoperative image of Case 1, and image 1B  depicts the preoperative image of Case 2.

**Figure 2:**
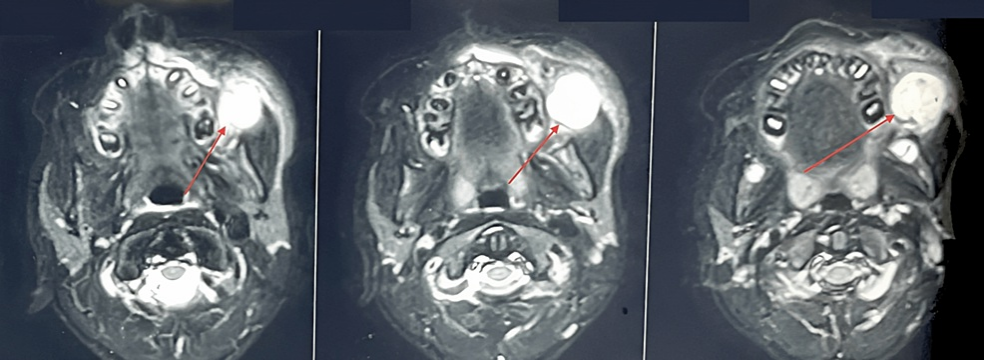
MRI showing the sections of the lesion in the left buccal mucosa The arrows in the MRI show a well-defined lesion in the left buccal mucosa measuring approximately 2x2.2x2.9cm. It can be noted that there is no bony involvement to be seen.

**Figure 3:**
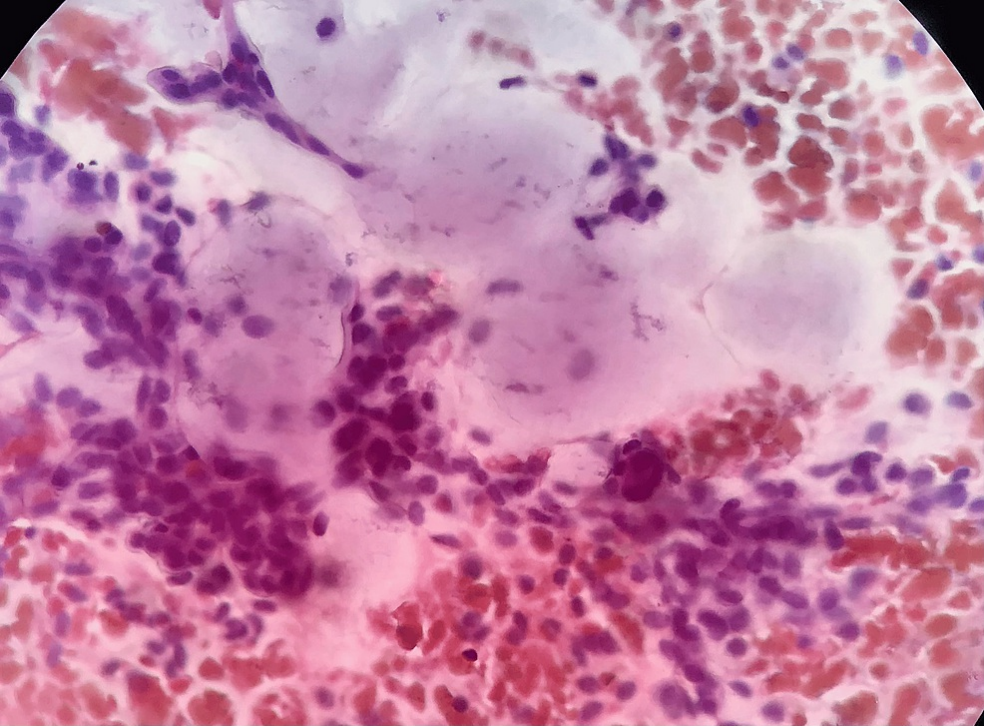
Fine needle aspiration cytology showing numerous areas of eosinophilic globular material suggestive of hyaline globules (MGG, 10x)

**Figure 4:**
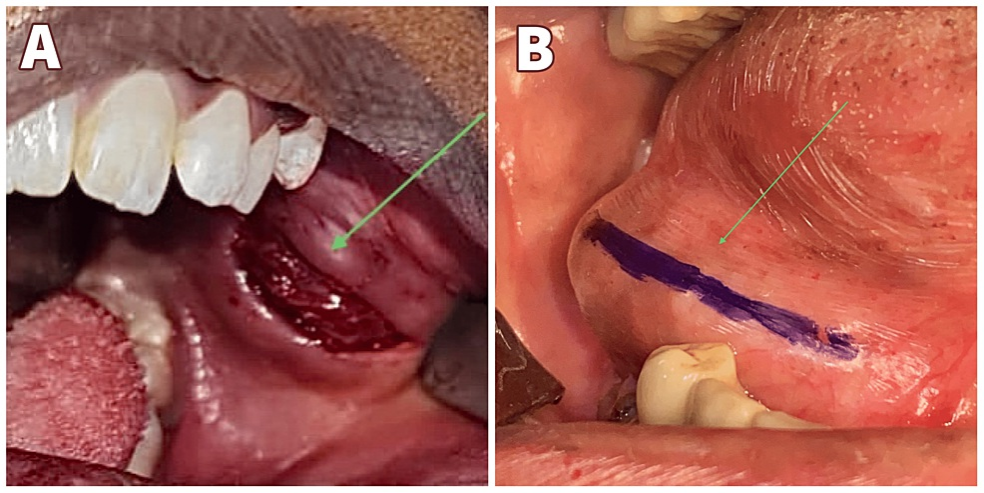
Intraoperative image showing the incision. In the above intraoperative image, the arrow in 4A represents the intraoral fibrotomy incision given in Case 1, and the arrow in 4B represents the incision marking in Case 2.

**Figure 5:**
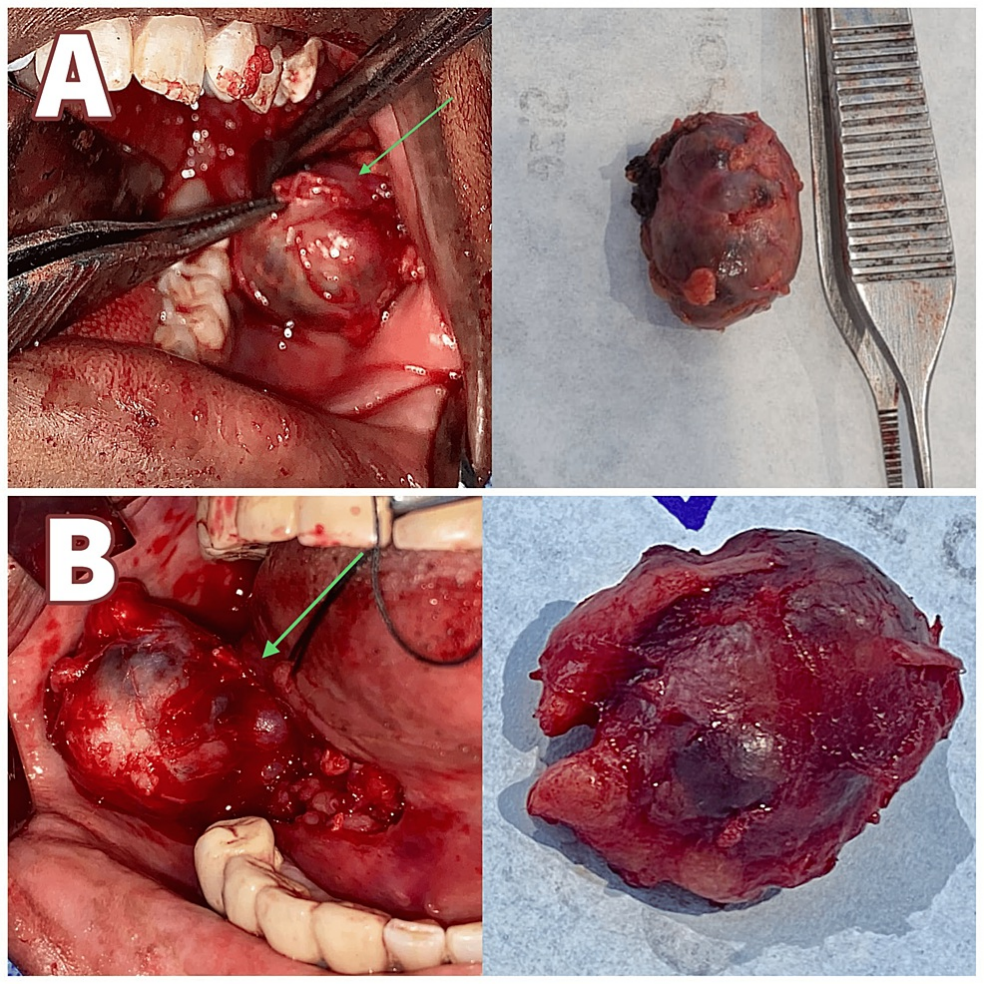
Intraoperative image showing the dissection and the excision of the lesion. In the above intraoperative image, the arrow in 5A represents the dissection (Left) and excised lesion (Right) of Case 1, and the arrow in 5B represents the dissection (Left) and excised lesion (Right) of Case 2.

**Figure 6:**
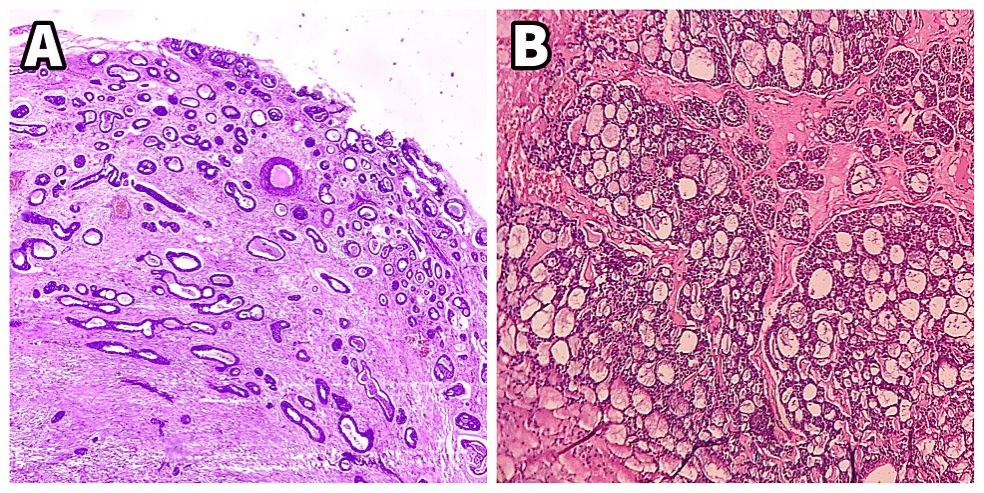
Histopathological slide of the excised specimen. In the above histopathological image, 6A represents the photomicrograph of excised specimen in Case 1, and 6B represents the photomicrograph of excised specimen in Case 2

An eight-year-old boy presented to the Department of Oral and Maxillofacial Surgery with a chief complaint of swelling on the left side of the face for over a year, showing signs of gradual increase over the past week. On examination, the patient had no constitutional symptoms but had swelling on the left side of the face involving the middle and the lower thirds with ambiguous borders leading to mild asymmetry. No surface ulcerations were seen, or temperature changes felt, and no regional lymphadenopathy. Intraorally a non-tender, soft to firm submucosal swelling of about 3x3 cm was palpated (Figure 1A). Based on the history and clinical findings, this swelling was provisionally diagnosed as a soft tissue neoplasm or a cystic swelling such as an Epidermal inclusion cyst, Fibrous tumor, etc. Intraoral Cone beam computed Tomography (CBCT) was taken, and it showed that there was no bone or dental involvement. MRI was advised based on the clinical features of soft tissue swelling without any bony or dental involvement. MRI revealed the lesion to be epi-centered in the left buccal and masticator space with no evidence of diffusion restriction and bony involvement suggestive of soft tissue pathology (Figure 2). FNAC was also done, pointing to a salivary gland lesion. The cytological smear stained section (Figure 3) shows numerous areas of eosinophilic globular material suggestive of hyaline globules admixed with predominantly round cells along with a few spindle-shaped cells and a few cells with an eccentrically placed nucleus. Mixed inflammatory cells infiltrate predominantly lymphocytes, and neutrophils are seen in a few areas. Few squamous cells and extravasated RBCs are also evident. Initially, an incisional biopsy was intended, but due to the uncooperative nature of the child and because the tumor had well-defined borders and was measured 2x2.2x2.9cm on MRI, we went ahead with excision under GA. An intraoral fibrotomy incision (Figure 4A) was made in the left buccal mucosa, and immediately after placing the incision, we encountered an encapsulated mass underneath, which was dissected free from the underlying buccinator muscle and mucosa (Figure 5A). Primary closure was achieved easily, and the post-operative period was uneventful. Post-operative histopathology with immunohistochemistry was done, and the results turned out to be suggestive of Grade 1 ACC with capsular invasion. Multiple sections showed a glandular, circumscribed, and encapsulated tumor mass comprising mainly coalescing strands of basaloid tumor cells within an extensively hyalinized connective tissue stroma. The tumor cells were arranged in the form of islands, sheets, nests, and tubules, which were polygonal to ovoid with deeply basophilic angular nuclei and scanty cytoplasm with well-delineated borders. Few tumor islands were punctuated by multiple rounds to oval spaces, often containing amphophilic to basophilic material, giving a "Swiss Cheese pattern" like arrangement. There was also evidence of tumor cells arranged in bilayered tubular structures. The central portion of the tumor mass showed extensive deposition of coalesced eosinophilic globules leading to the strangulation of tumor cells. There was also evidence of capsular invasion by the tumor islands along with epitheliomatous arrangement (around the blood vessels). Tyrosine-like crystals with moderate chronic lymphoplasmacytic infiltrate, rich vascularity, and extensive areas of hemorrhage were also seen. (Figure 6A).

The patient was on regular follow-ups at three months intervals. The patient was satisfied after surgery and was very adherent to the follow-up protocol.

Case 2

**Figure 7:**
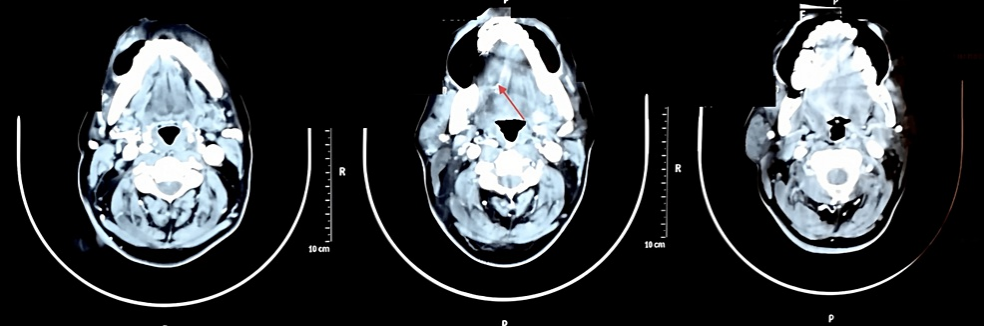
The CT with contrast image shows sections of the lesion in the right sublingual space. The arrow in the CT image shows a well-defined hypodense lesion measuring approximately 2.6x 1.5cm in the right sublingual space.

A 51-year-old female patient came to the Department of Oral and Maxillofacial Surgery with a chief complaint of swelling below the right side of the tongue for the past one year, and it increased in size in the past three months. The patient was normal three months back, after which there was a swelling below the tongue, which was small initially and progressed to the present size. On examination, blueish-colored sessile swelling below the tongue on the right side was noted, measuring 3x3 cm. It was not fluctuating, soft to firm in consistency painless. Mucosa was normal consistency (Figure 1B). The patient already had the reports of Computed tomography and ultrasound for the lesion. Computed Tomography (CT) revealed a well-defined ovoid hypodense lesion of ~2.6 x1.5 cm in the right sublingual space (Figure 7). Post-contrast administration shows a central non-enhancing component with mild peripheral enhancement. It is seen as causing indentation of the right genioglossus muscle medially. No evidence of adjacent bone erosion. No evidence of extension into submandibular space. Features suggest a benign cystic lesion in the right sub-lingual space (floor of mouth), likely Ranula (Figure 2B). Ultrasonographic reports revealed a well-defined thick-walled cystic lesion measuring ~2.5 x1.9 x1.5 cm with few internal echoes noted in the right sublingual space extending partly to the submandibular space without significant vascularity of the cyst wall. Features suggestive of Ranula - probably infected. Fine Needle Aspiration Cytology (FNAC) was not done given preventing the seeding of tumor cells as we also had minor salivary gland tumors as a differential diagnosis even though CT and USG were in favor of Ranula. We had provisionally diagnosed it as a Ranula or Sublingual salivary gland tumor of the right sublingual gland. Under general anesthesia, nasotracheal intubation was done, and standard scrubbing and draping were done. 2% lignocaine without adrenaline was given as local infiltration about the sublingual gland. The incision was placed on the lesion (Figure 4B), dissection around the lesion was done around the lesion, and the lesion was removed in toto, along with the sublingual gland (Figure 5B). The lesion was solid in consistency which proved it not to be a ranula. The closure was done using 3-0 vicryl (Polyglactin). Hemostasis achieved. Extubation was uneventful. The excised specimen was sent for histopathological examination. The histopathological report revealed Grade 1 Adenoid cystic carcinoma. Multiple sections showed an encapsulated, partially circumscribed neoplasm of salivary gland origin comprising mainly coalescing strands of basaloid tumor cells within a hyalinized connective tissue stroma. In a few areas, the tumor cells were seen invading the capsule. The tumor cells were arranged predominantly in a cribriform pattern, along with a few islands and nests. Tumor cells were polygonal to ovoid in shape with deeply basophilic angular nuclei and scanty cytoplasm with indistinct cell outlines. The tumor Islands were punctuated by numerous cystic spaces with basophilic material giving a Swiss cheese pattern and extensive areas of eosinophilic deposition around the tumor Islands. There was evidence of bilateral tubular structure with low columnar to cuboidal tumor cells. Hyalinized eosinophilic stroma was evident, with fewer tumor cells seen strangulated within the stroma. Moderate chronic inflammatory cell infiltrate and rich vascularity with an extensive area of hemorrhage were also seen. Numerous nerve bundles, minor salivary glands, and ducts are also evident (Figure 6B). Post-operative healing was uneventful. A regular follow-up for six months was done. No signs of recurrence were noted.

## Discussion

Adenoid cystic carcinoma is the second most commonly occurring malignant salivary gland tumor of the major and minor salivary glands. It has shown numerous clinical variations, which makes accurate diagnosis and appropriate treatment approach difficult for clinicians. The most common age of occurrence is in the fifth to sixth decade of life. These tumors are very uncommon in children [6]. Even though a few authors, such as Evesson and Cawson, discovered a female predilection, most researchers in the literature reported an equal distribution in both genders [7]. When it comes to etiology, there is no universally accepted etiology for adenoid cystic carcinoma. Usually, the tumor does not present with symptoms until it grows considerably, and patients report only after the lesion has invaded locally.

The most commonly employed radiographic investigation is CT. It helps delineate the extent of the lesion and can be used for inspecting any recurrence postoperatively. Ultrasonography can also be used for diagnosis, which was used in our second case. MRI can be used in soft tissue lesions, which helps to measure the exact size location of the lesion and if it has invaded locally. Various molecular studies have been made for advances in diagnosing salivary gland pathologies [8-10].

The final confirmatory diagnosis is given by histological examination. It not only helps in diagnosis, but the grading and the histological variant help to decide the appropriate treatment and the prognosis of the lesion [11]. Among the three histological types of this tumor, the solid variant is considered the most aggressive and needs extensive treatment planning and regular follow-ups [5].

The most widely accepted treatment is surgical excision of the lesion, and when there is distant metastasis, postoperative radiation is advised. The clinical course of the disease is very unpredictable, and the prognosis is poor in patients with perineural invasion, solid histological type, distant metastasis, and local recurrence. When the lesion invades the adjacent bone, there is a high chance of recurrence [5,12]. Various rare cases need special attention in diagnosis and treatment planning [13-17], and this can be done by knowledge sharing through the publication of the experiences as case reports, case series, randomized controlled trials, etc. [18-20].

In our first case, it occurred in an 8-year-old boy, and this is considered to be very rare due to the age of occurrence [4]. And it occurred in the buccal mucosa, which is also not a common site of occurrence. Usually, adenoid cystic carcinoma of the minor salivary glands usually occurs in the palate. Since it is an unusual presentation, adenoid cystic carcinoma was not one of the provisional diagnoses. Only after appropriate investigations we concluded.

In our second case, it occurred in a 50-year-old female patient. This is a typical presentation of adenoid cystic carcinoma considering the age and gender of occurrence. The only peculiar thing that misleads the diagnosis is the site of the tumor. It was present in the sublingual gland, which made us provisionally diagnose it as a ranula, and also, it was blueish, resembling a ranula. In both cases, the radiologist ruled out the perineural invasion.

There is a dearth of literature about pediatric adenoid cystic carcinoma management. As local lymphatic dissemination is uncommon, but the hematogenous spread is conceivable, the aim of therapy for individuals with ACC is local and distant control of the disease and preservation of function.

## Conclusions

Adenoid cystic carcinoma is a very unpredictable tumor occurring in the 5th to 6th decade in the major and minor salivary gland tumors. From the above-explained cases, it can be concluded that appropriate diagnostic measures, proper diagnosis, and prompt treatment help in the success of the surgery and prognosis of the disease. Even though such cases occur in rare instances, we, as Oral and Maxillofacial surgeons, need awareness regarding such lesions to provide proper patient care.
